# Phylogenetic Analysis of the *Bifidobacterium* Genus Using Glycolysis Enzyme Sequences

**DOI:** 10.3389/fmicb.2016.00657

**Published:** 2016-05-09

**Authors:** Katelyn Brandt, Rodolphe Barrangou

**Affiliations:** ^1^Functional Genomics Graduate Program, North Carolina State UniversityRaleigh, NC, USA; ^2^Department of Food, Bioprocessing and Nutrition Sciences, North Carolina State UniversityRaleigh, NC, USA

**Keywords:** *Bifidobacterium*, glycolysis, phylogeny, probiotic, evolution

## Abstract

Bifidobacteria are important members of the human gastrointestinal tract that promote the establishment of a healthy microbial consortium in the gut of infants. Recent studies have established that the *Bifidobacterium* genus is a polymorphic phylogenetic clade, which encompasses a diversity of species and subspecies that encode a broad range of proteins implicated in complex and non-digestible carbohydrate uptake and catabolism, ranging from human breast milk oligosaccharides, to plant fibers. Recent genomic studies have created a need to properly place *Bifidobacterium* species in a phylogenetic tree. Current approaches, based on core-genome analyses come at the cost of intensive sequencing and demanding analytical processes. Here, we propose a typing method based on sequences of glycolysis genes and the proteins they encode, to provide insights into diversity, typing, and phylogeny in this complex and broad genus. We show that glycolysis genes occur broadly in these genomes, to encode the machinery necessary for the biochemical spine of the cell, and provide a robust phylogenetic marker. Furthermore, glycolytic sequences-based trees are congruent with both the classical 16S rRNA phylogeny, and core genome-based strain clustering. Furthermore, these glycolysis markers can also be used to provide insights into the adaptive evolution of this genus, especially with regards to trends toward a high GC content. This streamlined method may open new avenues for phylogenetic studies on a broad scale, given the widespread occurrence of the glycolysis pathway in bacteria, and the diversity of the sequences they encode.

## Introduction

*Bifidobacterium* species are an important component of the human gastrointestinal tract (GIT) microbiome, and exert critical functional roles, especially during the establishment of gut microbial composition early in life. Consequently, they are the subject of extensive microbiological and genetics studies, to investigate their probiotic phenotypes, and genotypes, respectively. Actually, many studies are investigating the genetic basis for their health-promoting functionalities, both in industry and academia. This genus is often found in the GIT of animals (Ventura et al., 2014), and is the predominant phylogenetic group early in human life (Turroni et al., 2012a). Indeed, a mounting body of evidence has established vertical transmission between the mother and infants (Milani et al., 2015), notably through the selective nurture of bifidobacteria through diverse non-digestible human-milk oligosaccharides (HMOs) that are a critical component of breast milk (Sela, 2011). These HMOs selectively drive the colonization of the infantile GIT by species that encode prebiotic transporters and hydrolases (Turroni et al., 2012b). Recently, a dichotomy has been established between healthy term babies with a normal gut microbiome, and preterm infants whom have not been colonized by *Bifidobacterium* species (Arboleya et al., 2015). Several studies have implicated the expansive carbohydrate uptake and catabolism gene repertoire of bifidobacteria as the key driver of adaptation of this genus to the infant diet (Milani et al., 2014). In fact, several species of bifidobacteria have shown unique genome composition adaptation trajectories in their carbohydrate utilization machinery, rendering them competitive in this environment (Pokusaeva et al., 2011; Ventura et al., 2012).

To better understand how these organisms have emerged as potent early-life colonizers, there has been a surge in genome sequencing in recent years. At the time of writing, 47 established species and subspecies have been sequenced (Milani et al., 2016), providing a wealth of genomic information, which serves as a valuable tool for understanding the species and strain diversity within this polymorphic genus, as well as unraveling the key elements that drive health-promoting and colonization phenotypes in humans. However, given the democratization of sequencing technologies in general, and genome and microbiome sequencing in particular, it is imperative that tools and methods be available to analyze this high-throughput data, and specifically allow experimentalists to parse out the complex phylogeny of this broad genus. Indeed, basic questions being addressed regarding the occurrence, diversity and functions of various *Bifidobacterium* species in the human GIT will require the ability to accurately and consistently assign phylogeny.

Fundamentally, as new sequences become available, it is important to know where to place strains on the phylogenetic tree of *Bifidobacterium.* Whereas the affordability, accessibility and ability to generate high-throughput data have become somewhat straightforward, a key challenge lies in the analysis of these sequences, regarding assembly, comparative analyses and phylogenetic assignments. Historically, 16S rRNA sequences have been used across the phylogenetics field for classification and sequence tree-based assignments, but there are growing concerns about the adequacy and sustainability of this method (Fox et al., 1992), notably with regards to the availability of proper references (Clarridge, 2004), and the actual levels of conservation of sequences targeted by “universal” primers (Baker et al., 2003). Because of this, new approaches have been suggested, ranging from multi-locus approaches, using housekeeping genes (Eisen, 1995), to core-genome analyses (Medini et al., 2005). For *Bifidobacterium*, efforts have been focused on creating a phylogeny based on whole and/or conserved genomic sequences, namely the pan-genome and the core-genome, respectively (Lukjancenko et al., 2011; Lugli et al., 2014). While the core-genome is arguably comprehensive, core-genome assembly is time consuming and computationally intense. Alternative methods need to be developed, to allow rapid and convenient phylogenetic screening of new and potentially unknown sequences. Preferably, such a method would provide high resolution, low-throughput, robust, accurate, and affordable information.

Notwithstanding phenotypic diversity between organisms that have specialized metabolic pathway combinations, and the corresponding genomic complement, there are core biochemical pathways and processes that are broadly distributed across the Tree of Life. Noteworthy, glycolysis is a fundamental process for most cells, and may be construed as the biochemical backbone of most, if not all, living organisms. Indeed, this process allows for the genesis of energy through the catabolism of simple carbohydrates. This pathway is, at least partially, present in all genomes (Fothergill-Gilmore and Michels, 1993) and consequently constitutes a promising biochemical, and thus genetic, marker for phylogenetic studies. Because these genes are important, they are typically members of the house-keeping genomic set, and are widely dispersed across the Tree of Life. However, they are likely subject to less selective pressure than other phylogenetic markers (i.e., ribosomal sequences), and thus afford a more diverse set of sequences to encompass a broad range of assorted sequences (Fothergill-Gilmore, 1986). Therefore, we set out to assess the potential of glycolytic genes, and the sequences of the proteins they encode, for bifidobacteria phylogenetic studies. In particular, we determined the occurrence and diversity of these glycolytic enzyme genes in the genomes of bifidobacteria, and compared and contrasted sequence alignment-based trees with one another, and to those derived from alternative sequences, notably the core-genome, and the 16S rRNA-based reference tree. Our results show how the glycolysis protein sequences can be used as suitable markers to create a phylogeny of *Bifidobacterium* that is as accurate as the core-genome based phylogeny, but much less computationally demanding. We also explore how basic features of the genetic sequences of glycolysis can reveal trends and patterns of evolution among the different *Bifidobacterium* species and the genus as a whole.

## Materials and Methods

### Genetic Sequences Sampling and Reference Genomes

We used sequences derived from a total of 48 *Bifidobacterium* genomes from distinct species and subspecies, as listed in **Table 1**. *Bifidobacterium stercoris* was included in this analysis, as a separate species, but it was recently renamed as a strain of *Bifidobacterium adolescentis* (Killer et al., 2013). Our results (see below) show that *B. stercoris* is always a close neighbor of *B. adolescentis*, consistent with the newest findings. These genomes were mined for the presence of glycolytic enzymes using Geneious version 9.0.5(Kearse et al., 2012). We selectively elected to pursue a scheme based on canonical glycolysis genes, as to generate a broadly applicable method. Nevertheless, the classical glycolysis genes do not universally occur in bacterial genomes. Furthermore, some organisms do carry alternative pathways, such as the bifid shunt in *bifidobacterium*, which could prove valuable, but are not widely distributed. The nine canonical glycolysis enzymes from bifidobacteria (de Vries and Stouthamer, 1967) were found in each genome. Four reference species (*Bifidobacterium longum* subsp. *longum*, *B. adolescentis*, *Bifidobacterium animalis sub. lactis*, and *Bifidobacterium breve*) were used to make a database of the nine genes. The Annotate from Database feature was used (with 40% nucleotide sequence similarity cut-off) to identify glycolytic orthologs in the other genomes. As all genomes had been previously annotated, we confirmed the original annotation to the database annotation manually to validate this method of mining. In cases where multiple hits were obtained, BLAST (Altschul et al., 1990) analyses were carried out to select the correct homolog. Translated sequences were confirmed using ExPasy (Gasteiger et al., 2003). For the 16S rRNA analysis, the 16S rRNA sequences were extracted manually from each genome. In case of multiple hits, BLAST analyses were carried out to select the right sequences. For increased robustness, the glycolysis enzyme sequences were concatenated in order of occurrence in the glycolysis pathway (Lang et al., 2013).

**Table 1:** Species and genome list.

Genus	Species	Subspecies	Strain	Accession number	Naming convention	Locus tag
*Bifidobacterium*	*actinocoloniiforme*		DSM 22766	NZ_CP011786	B_actinocoloniiforme	AB656
*Bifidobacterium*	*adolescentis*		ATCC 15703	NC_008618	B_adolescentis	BAD


*Bifidobacterium*	*angulatum*		LMG 11039	NZ_JGYL00000000	B_angulatum	BIANG


*Bifidobacterium*	*animalis*	*animalis*	ATCC 22527	NC_017834	B_animalis_a	BANAN


*Bifidobacterium*	*animalis*	*lactis*	DSM 10140	NC_012815	B_animalis_l	BALAT


*Bifidobacterium*	*asteroides*		PRL 2011	NC_018720	B_asteroides	BAST


*Bifidobacterium*	*biavatii*		DSM 23969	NZ_JDUU00000000	B_biavatti	OU23


*Bifidobacterium*	*bifidum*		LMG 13200	NZ_JSEB00000000	B_bifidum	LMG13200


*Bifidobacterium*	*bohemicum*		DSM 22767	NZ_JDUS00000000	B_bohemicum	OU21


*Bifidobacterium*	*bombi*		DSM 19703	NZ_JDTS00000000	B_bombi	OT95


*Bifidobacterium*	*boum*		LMG 10736	NZ_JGYQ00000000	B_boum	BBOU


*Bifidobacterium*	*breve*		UCC 2003	NC_020517	B_breve	Bbr


*Bifidobacterium*	*callitrichos*		DSM 23973	NZ_JGYS00000000	B_callitrichos	BCAL


*Bifidobacterium*	*catenulatum*		JCM 1194	NZ_AP012325	B_catenulatum	BBCT


*Bifidobacterium*	*choerinum*		LMG 10510	NZ_JGYU00000000	B_choerinum	BCHO


*Bifidobacterium*	*coryneforme*		LMG 18911	NZ_CP007287	B_coryneforme	BCOR


*Bifidobacterium*	*crudilactis*		LMG 23609	NZ_JHAL00000000	B_crudilactis	DB51


*Bifidobacterium*	*cuniculi*		LMG 10738	NZ_JGYV00000000	B_cuniculi	BCUN


*Bifidobacterium*	*dentium*		Bd1	NC_013714	B_dentium	BDP


*Bifidobacterium*	*gallicum*		DSM 20093	NZ_ABXB00000000	B_gallicum	BIFGAL


*Bifidobacterium*	*gallinarum*		LMG 11586	NZ_JGYX00000000	B_gallinarum	BIGA


*Bifidobacterium*	*indicum*		LMG 11587	NZ_CP006018	B_indicum	BINDI


*Bifidobacterium*	*kashiwanohense*		JCM 15439	NZ_AP012327	B_kashiwanohense	BBKW


*Bifidobacterium*	*longum*	*longum*	NCC 2705	NC_004307	B_longum	BL


*Bifidobacterium*	*longum*	*infantis*	ATCC 15697	NC_011593	B_longum_i	Blon


*Bifidobacterium*	*longum*	*suis*	LMG 21814	NZ_JGZA00000000	B_longum_s	BLSS


*Bifidobacterium*	*magnum*		LMG 11591	NZ_JGZB00000000	B_magnum	BMAGN


*Bifidobacterium*	*merycicum*		LMG 11341	NZ_JGZC00000000	B_merycicum	BMERY


*Bifidobacterium*	*minimum*		LMG 11592	NZ_JGZD00000000	B_minimum	BMIN


*Bifidobacterium*	*mongoliense*		DSM 21395	NZ_JGZE00000000	B_mongoliense	BMON


*Bifidobacterium*	*moukalabense*		DSM 27321	NZ_AZMV00000000	B_moukalabense	BMOU


*Bifidobacterium*	*pseudocatenulatum*		JCM 1200	NZ_AP012330	B_pseudocatenulatum	BBPC


*Bifidobacterium*	*pseudolongum*	*globosum*	LMG 11569	NZ_JGZG00000000	B_pseudolongum_g	BPSG


*Bifidobacterium*	*pseudolongum*	*pseudolongum*	LMG 11571	NZ_JGZH00000000	B_pseudolongum_p	BPSP


*Bifidobacterium*	*psychraerophilum*		LMG 21775	NZ_JGZI00000000	B_psychraerophilum	BPSY


*Bifidobacterium*	*pullorum*		LMG 21816	NZ_JGZJ00000000	B_pullorum	BPULL


*Bifidobacterium*	*reuteri*		DSM 23975	NZ_JGZK00000000	B_reuteri	BREU


*Bifidobacterium*	*ruminantium*		LMG 21811	NZ_JGZL00000000	B_ruminantium	BRUM


*Bifidobacterium*	*saeculare*		LMG 14934	NZ_JGZM00000000	B_saeculare	BSAE


*Bifidobacterium*	*saguini*		DSM 23967	NZ_JGZN00000000	B_saguini	BISA


*Bifidobacterium*	*scardovii*		LMG 21589	NZ_JGZO00000000	B_scardovii	BSCA


*Bifidobacterium*	*stellenboschense*		DSM 23968	NZ_JGZP00000000	B_stellenboschense	BSTEL


*Bifidobacterium*	*stercoris*		DSM 24849	NZ_JGZQ00000000	B_stercoris	BSTER


*Bifidobacterium*	*subtile*		LMG 11597	NZ_JGZR00000000	B_subtile	BISU


*Bifidobacterium*	*thermacidophilum*	*porcinum*	LMG 21689	NZ_JGZS00000000	B_thermacidophilum_p	BPORC


*Bifidobacterium*	*thermacidophilum*	*thermacidophilum*	LMG 21395	NZ_JGZT00000000	B_thermacidophilum_t	THER5


*Bifidobacterium*	*thermophilum*		JCM 7027	-	B_thermophilum	BTHER


*Bifidobacterium*	*tsurumiense*		JCM 13495	NZ_JGZU00000000	B_tsurumiense	BITS



*List of the 48 species and subspecies used in this study. Accession numbers and naming conventions included*.

### Genesis of Sequence Alignment-based Trees

Five different alignments were made for each tree using Geneious version 9.0.5. ClustalW (Larkin et al., 2007) was used, with the BLOSUM scoring matrix, and settings of gap creation at -10 cost, and gap extension at -0.1 cost per element. For the 16S rRNA alignment, ClustalW was set so that the cost matrix was IUB, with a gap opening penalty of 15, and gap extension cost of 6.66. MUSCLE (Edgar, 2004) was used with the setting of eight maximum number of iterations for the amino acid sequences and the 16S rRNA alignments. The Geneious Pairwise Alignment was set so that the alignment type was global alignment with free end gaps and the cost matrix was BLOSUM62 for the amino acid sequences. For the 16S rRNA gene analysis, the alignment type was global alignment with free end gaps and a cost matrix of 65% similarity (5.0/-4.0). MAFFT (Katoh et al., 2002) was used twice, for both the amino acid sequences and the 16S rRNA sequences. For the amino acid sequences the first alignment had an algorithm setting of auto, a scoring matrix of BLOSUM62, a gap open penalty of 1.53, and an offset value of 0.123. The second alignment had an algorithm setting of auto, a scoring matrix of BLOSUM80, a gap open penalty of 1.53, and an offset value of 0.123. For the first 16S rRNA alignment, the algorithm was set to auto, the scoring matrix was set to 100 PAM/k = 2, the gap open penalty was set to 1.53, and the offset value was set to 0.123. The second alignment for the 16S rRNA was set so that the algorithm was auto, the scoring matrix was 200 PAM/k = 2, the gap open penalty was 1.53, and the offset value was 0.123. trimAl (Capella-Gutiérrez et al., 2009) was used to select a consistent alignment between the five alignments. The parameters were compareset and automated1. Using Geneious, trees were made from the respective consistent alignments. The trees were generated using RaxML version 7.2.8 (Stamatakis, 2006b, 2014). For the protein based trees the parameters were set so that the model was CAT (Lartillot and Philippe, 2004) BLOSUM62, the algorithm was Bootstrap using rapid hill climbing with random seed 1, and the number of bootstrap replicates was 100 (Stamatakis, 2006a). For the 16S rRNA tree, the nucleotide model was GTR CAT, the algorithm was Bootstrap using rapid hill climbing with random seed 1, and the number of bootstrap replicates was 100. A consensus tree was then built using the consensus builder in Geneious, at a 50% support threshold. The consensus tree was used in all further analyses. The sums of branch lengths for each tree were found by adding the branch lengths together in Mega6 (Tamura et al., 2013).

### Statistical Analyses

All statistical analyses were carried out using R version 3.2.2 (R Core Team, 2015). This software was also used to generate plots, graphs and display quantitative data throughout the manuscript.

## Results

### Glycolytic Enzyme Sequence-based Phylogeny

**FIGURE 1:**
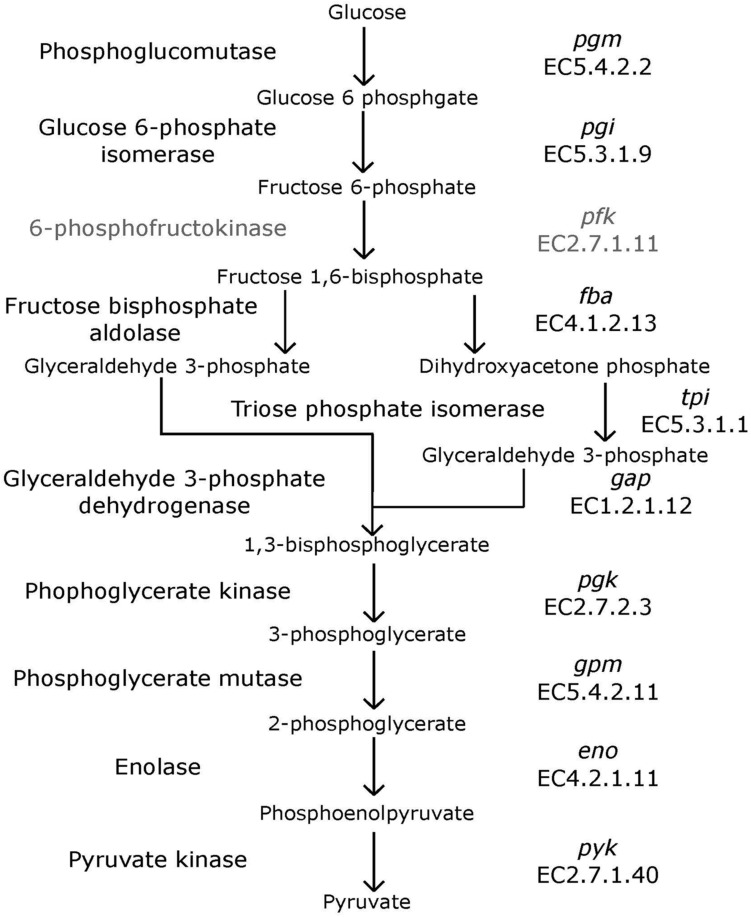
**Glycolysis pathway**. Traditional biochemical pathway of glycolysis. Enzyme names are listed to left of arrows, and gene names and EC numbers are shown on the right. 6-phosphofructokinase is faded to represent its absence in *Bifidobacterium*.

**FIGURE 2:**
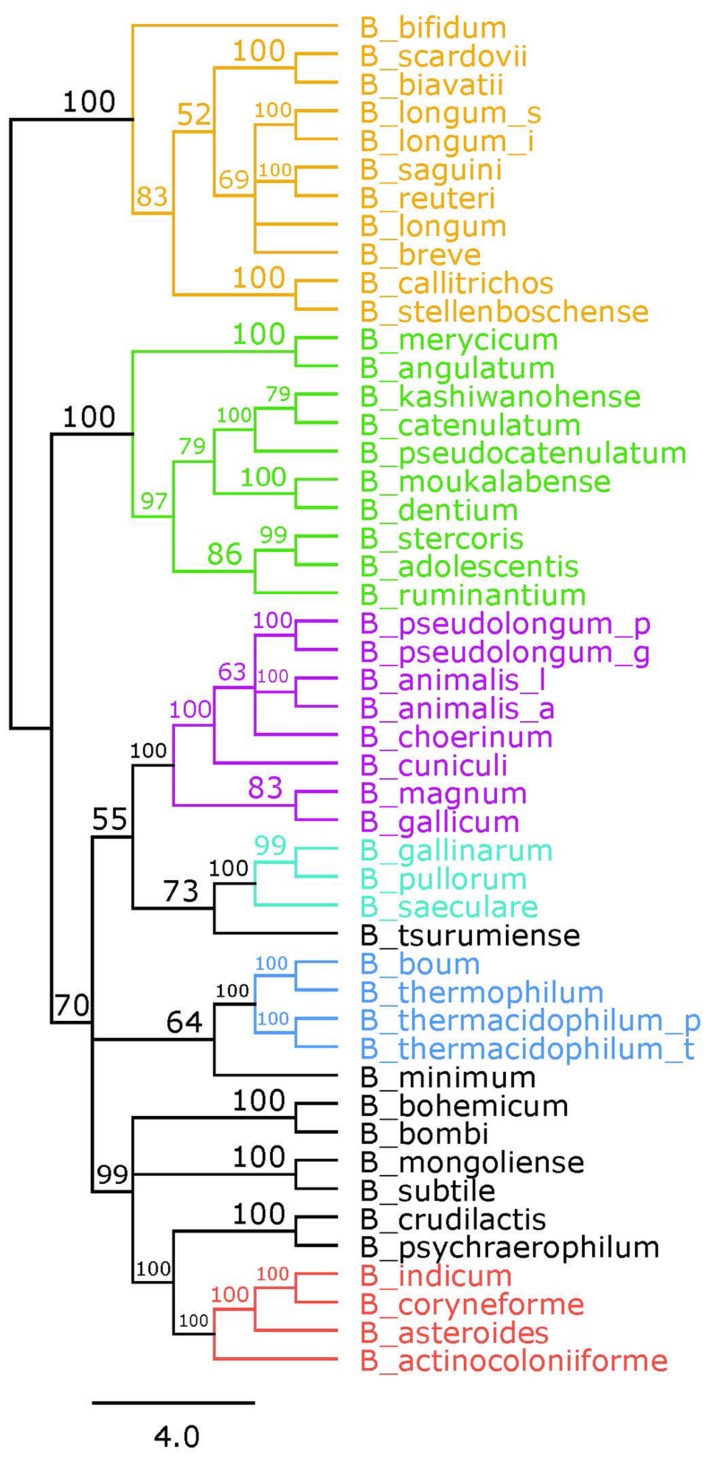
**Glycolytic proteins concatenated tree**. Consensus tree based on alignment of the concatenated amino acid sequences of the glycolysis pathway found in *Bifidobacterium*. Trees were made using RaxML. Bootstrap values are found on each node. Phylogenetic groups are colored as follows: *Bifidobacterium longum* is orange, *Bifidobacterium adolescentis* is green, *Bifidobacterium psdeudolongum* is purple, *Bifidobacterium pollorum* is blue-green, *Bifidobacterium boum* is blue, and *Bifidobacterium asteroides* is red. Species names follow the naming convention from **Table 1**.

Bifidobacteria contain nine of the 10 traditional enzymes (**Figure 1**) commonly found in the glycolysis pathway (de Vries and Stouthamer, 1967). Phylogenetic analyses were carried out using the amino acid sequences of the proteins encoded by the aforementioned glycolysis genes. A comprehensive tree based on sequence alignment of the concatenated sequences of the glycolytic enzymes found in *Bifidobacterium* is shown in **Figure 2**. Six separate phylogenetic groups were identified, as previously established from the core-genome (Milani et al., 2016). These groups are: the *B. longum* group (orange), the *B. adolescentis* group (green), the *Bifidobacterium pseudolongum* group (purple), the *Bifidobacterium pollurom* group (blue-green), the *Bifidobacterium boum* group (blue), and the *Bifidobacterium asteroides* group (red; Bottacini et al., 2014). The number of individuals in each group varied between 3 and 11, with the *B. longum* group being the most diverse. *Bifidobacterium angulatum* and *Bifidobacterium merycicum* were moved to the *B. adolescentis* group due to a high bootstrap value in the concatenated tree. The concatenated tree has bootstrap values that range from 52 to 100. We observe a total of 34 bootstrap values of 70 and above (Supplementary Figure S1). Trees based on sequence alignments of the individual enzymes of glycolysis can be found in Supplementary Figures S2–S10. Interestingly, all of the individual trees resolved the phylogenetic groups found in the core-genome with only the Gap and Eno trees providing alternative locations for a few branches, notably *Bifidobacterium magnum*, *Bifidobacterium gallicum*, and *Bifidobacterium thermacidophilum sub. thermacidophilum*. **Table 2** shows the sum of branch lengths for each tree. The 16S rRNA tree has the largest sum at 204.99, while the concatenated tree had the smallest sum at 99.56. The consistent clustering into these six phylogenetic trees illustrates how robust and valuable the glycolytic sequences are with regards to phylogenetic information. It also shows that this method is congruent with the core-genome.

**Table 2:** Sum of branch lengths for each tree.

Gene	E. C. number	Sum
Phosphoglucomutase (pgm,1)	5.4.2.2	125.03
Glucose-6-phosphate isomerase (pgi,2)	5.3.1.9	153.43
Fructose bisphosphate aldolase (fba, 4)	4.1.2.13	151.76
Triose phosphate isomerase (tpi, 5)	5.3.1.1	170.61
Glyceraldehyde 3-phosphate dehydrogenase (gap, 6)	1.2.1.12	103.07
Phsophoglycerate kinase (pgk, 7)	2.7.2.3	132.41
Phosphoglycerate mutase (gpm, 8)	5.4.2.11	174.7
Enolase (eno, 9)	4.2.1.11	145.06
Pyruvate kinase (pyk, 10)	2.7.1.40	107.56
Concatenated	-	99.56
16S rRNA	-	204.99

*Sum of branch lengths for each tree. EC number for each enzyme is also listed*.

### 16S rRNA-based Reference Phylogeny

**FIGURE 3:**
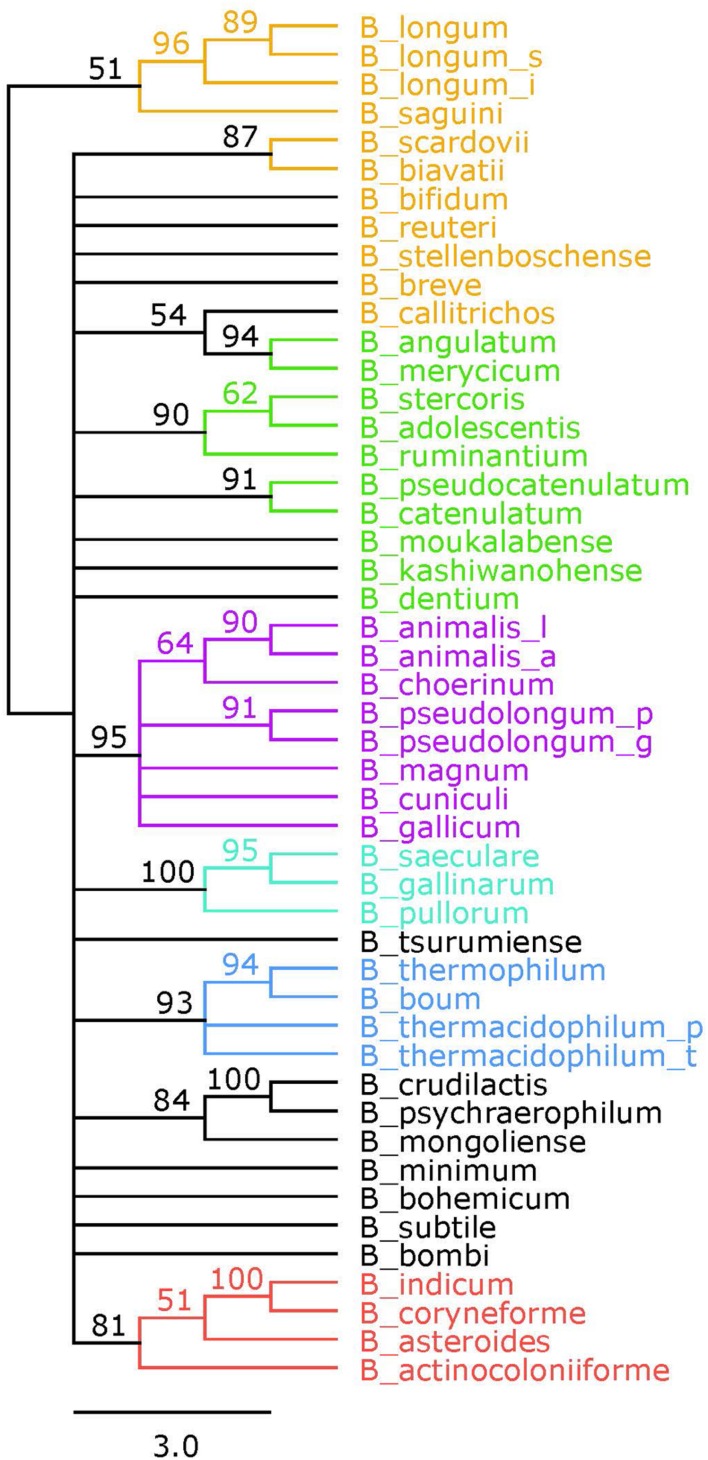
**16S rRNA phylogenetic tree**. Consensus tree based on alignment of the 16S rRNA sequences. Trees were made using RaxML. Bootstrap values are found on each node. Phylogenetic groups are colored as follows: *B. longum* is orange, *B. adolescentis* is green, *B. psdeudolongum* is purple, *B. pollorum* is blue-green, *B. boum* is blue, and *B. asteroides* is red. Species names follow the naming convention from **Table 1**.

A reference phylogeny was generated using the 16S rRNA sequences of each of the 48 species and sub-species included in this study (**Figure 3**). The six phylogenetic groups are identified and colored the same as in the concatenated tree. We elected to assign the *B. angulatum* and *B. merycicum* from the *B. longum* group to the *B. adolescentis* group, consistent with the concatenated tree. Noteworthy, the tree has bootstrap values that range from 51 to 100, with 17 nodes at values of 70 and above, which is half the amount found in the concatenated tree (Supplementary Figure S1). With regards to size, we point out that the concatenated tree is based on overall sequences ranging between 3,205 amino acids and 3,479 amino acids, which quantitatively compares as approximately twice the amount to the 16S rRNA ∼1,600 nt range, in terms of input-information amounts.

### Genome-Wide Analyses

**FIGURE 4:**
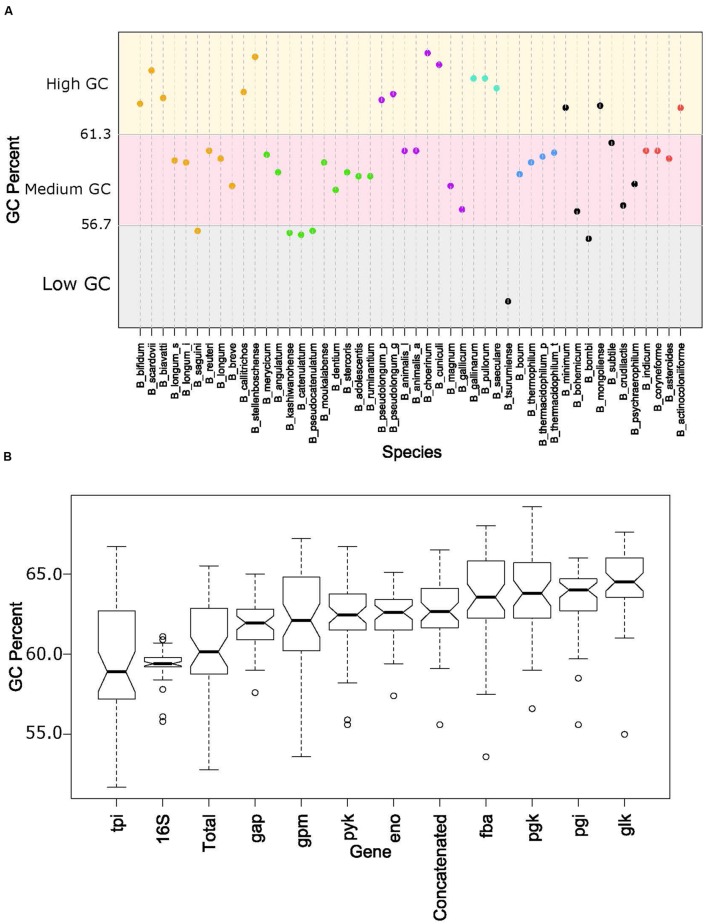
**GC content by species and glycolytic genes**. **(A)** Shows the total GC content of each species organized by the glycolytic concatenated tree. Spectrum is split into three groups: low GC from 0.52 to 0.567 (gray), Medium GC from 0.567 to 0.613 (pink), and High GC from 0.613 to 0.66 (yellow). Phylogenetic groups are colored as follows: *B. longum* is orange, *B. adolescentis* is green, *B. psdeudolongum* is purple, *B. pollorum* is blue-green, *B. boum* is blue, and *B. asteroides* red. Species names following the naming convention from **Table 1**. **(B)** contains notched boxplots of the GC values of each gene and total GC. Boxes are ranked in order of median. Notches that do not overlap are indicative of strong evidence of difference between two medians.

The overall genome sizes in this study ranged from 1.73 Mb for *Bifidobacterium indicum* to 3.26 Mb for *Bifidobacterium biavatii*, with an average of 2.28 Mb and a median of 2.17 Mb. The GC content ranged from 52.8% for *Bifidobacterium tsurumiense* to 65.5% for *Bifidobacterium choerinum*, with an average of 60.4% and a median of 60.2%. This substantiates the perception that bifidobacteria are generally categorized as high-GC content organisms, at the genome-wide level (Ventura et al., 2007). However, a thorough analysis of GC content across the phylogenetic groups revealed that even among these high-GC organisms there are three distinct subsets of high, medium, and low-GC bifidobacteria (**Figure 4A**). Most of the species fall in the upper medium-GC range, with the low-GC range being the least populated. There are some noteworthy groupings between the phylogenetic groups, specifically the *B. pullorum* and the *B. boum* groups, for which the entire groups are packed tightly in the high GC region and the medium GC region, respectively. All of the other groups, except the *B. longum* group, span two of these subsets. For the *B. longum* group, *Bifidobacterium saguini* lies just at the border between the low and medium GC subsets. This group has the largest spread, consistent with being the most diverse in the concatenated and 16S rRNA trees.

**FIGURE 5:**
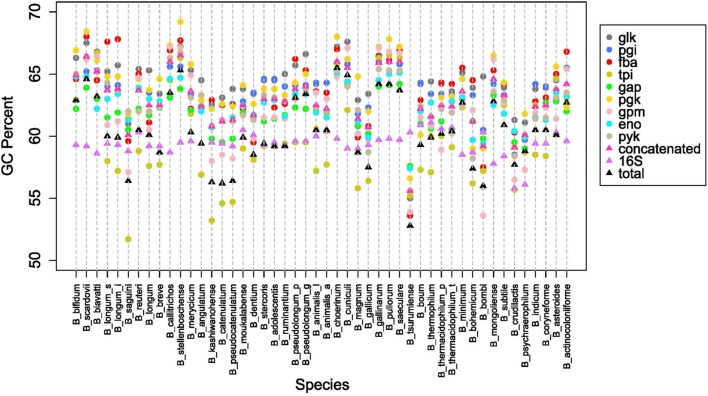
**Overall GC content patterns across species**. GC percent for each glycolysis gene, 16SrRNA and overall genome, for species listed in **Table 1**.

Next we looked at how the GC content varied across the trees. **Figure 4B** shows boxplots of the GC content of each tree and the total GC content. Except for the 16S rRNA and *tpi* trees, all other trees had median GC values with strong evidence of being higher than the median total GC content (Chambers, 1983). Looking on an individual basis, over half of the genomes have 16S rRNA and *tpi* GC values below their total GC, while the other genes are either above or close to their total GC (**Figure 5**). Again, the *B. pullorum* and *B. boum* groups are tightly packed in regards to their GC spread amongst their glycolysis genes, 16S rRNA, and total GC. In contrast, the *B. longum* group has the largest spread, a parallel to its higher diversity in the phylogenetic trees.

## Discussion

*Bifidobacterium* is a diverse genus of human intestinal beneficial microbes that provide health-promoting functionalities, as illustrated by their broad use as probiotics in foods and dietary supplements (Turroni et al., 2014). Recently, extensive genomic analyses of diverse species, subspecies and phylogenetic groups have provided insights into their adaptation to the human gut, notably with regards to their ability to colonize the intestinal cavity in general, and utilize non-digestible carbohydrates in particular (Milani et al., 2016). Studies investigating the use of human breast milk oligosaccharides illustrate the important contribution of these probiotics in establishing the human gut microbiome at the early stages of life (Sela, 2011). Yet, these studies also reveal that there are many distinct and diverse *Bifidobacterium* species and phylogenetic groups that colonize the human GIT, perhaps with idiosyncratic genomic attributes, and their corresponding functionalities (Chaplin et al., 2015). These organisms have specifically adapted to their environment to competitively utilize available nutrients (Sánchez et al., 2013). In the human gut, these consist of non-digestible complex oligosaccharides that are not adsorbed, nor broken down in the upper GIT. Whereas, plant-based fibers are important in the adult diet, HMOs are important components of the infant diet. Furthermore, *Bifidobacterium* have even been successful in helping each other through cross-feeding (Turroni et al., 2015). Thus, we addressed the need to establish practical means to allocate phylogeny with minimalistic information based on sequences that encode glycolysis, the biochemical spine of most cells.

Here, we have shown that a multigene approach using glycolysis sequences can be used to uncover genomic trends and to make an accurate phylogenetic tree, based on a relatively small amount of information. The concatenated glycolysis tree in **Figure 2** is congruent with both the 16S rRNA tree and the established core-genome-based tree (Milani et al., 2016). The only notable exception is the placement of *B. merycicum* and *B. angulatum*. However, the relocation was between two neighboring phylogenetic groups in the concatenated and core-genome based trees. The glycolysis pathway is perhaps as, if not more, robust and accurate than the 16S rRNA tree. Compared to the 16S rRNA, the bootstrap values of the concatenated tree were higher on average. This leads to more confidence in the placement of species and the identification of phylogenetic groups, which in comparison, can appear arbitrarily located on the 16S rRNA. The concatenated tree is able to identify groups as well as the core-genome based tree. In fact, all of the phylogenetic groups from the core-genome were consistently found across the glycolytic pathway based trees. However, the glycolysis-based trees have the advantage of being much less labor intensive than the core-genome approach. This allows for accurate phylogenetic mapping of new strains or species, possibly encompassing unknown species, in less time and with less data than a core-genome. This approach is high resolution, low throughput, affordable, and accurate. Part of the success of this approach is the universality of glycolysis. Glycolysis is the biochemical backbone of the cell, and as such all organisms have at least some part of the glycolysis pathway represented (Fothergill-Gilmore and Michels, 1993). Even though these are slower-evolving genes, the changes that are made are enough to make an accurate phylogeny (Fothergill-Gilmore, 1986), evidenced from the congruence between our trees and the core-genome based tree. Even though the glycolysis enzymes are considered “slow evolvers,” our data shows they are evolving at different rates amongst themselves. This can be explained by the fact that the glycolysis pathway is adapted by organisms to best fit their own unique environment and requirements (Bar-Even et al., 2012), as seen here in the *Bifidobacterium* and their bifid shunt (Sela et al., 2010). Some of the genes have specialized secondary functions, such as enolase acting as a cell surface receptor in *Bifidobacterium* (Candela et al., 2009*).* All of this makes the glycolysis pathway an excellent phylogenetic marker candidate. The various rates in evolution and moonlighting abilities also allow for further applications in recognizing adaptive trends.

The functional diversity of bifidobacteria is underpinned by multi-dimensional variety in their genomes, including overall content, organization, sequence diversity, and others. In extreme cases, even a two-fold difference in genome size can be observed. Despite being generally perceived as high GC organisms, they vary enough to have distinct relative classes of high, middle, and low-GC, amongst themselves (**Figure 4A**). Yet, there are non-random patterns and phenomena that drive these differences. The phylogenetic groups are clustered in specific regions of the GC continuum. Some groups are more tightly packed than others. A general trend that is observed across the genus is an evolutionary movement toward a high(er) GC content. The higher end of the spectrum is more densely populated then the lower end of the spectrum, indicative of an upward trend. This is reflected by the increased GC content in the individual glycolysis genes, when compared to the total GC content. Of the glycolysis genes, only one, *tpi*, does not show strong evidence for being different from the genome-wide (total) GC content. Critically, all of the other genes are above the total GC content. When we combine the overall genomic data with the GC-content groupings and trends discovered using glycolysis as phylogenetic markers, we posit the hypothesis that, over time, the GC content within the genomes of bifidobacteria increases, as to deviate further away from the 50% value, as the organisms adapt, and their genomes evolve accordingly.

Because of the broad occurrence of the glycolysis pathway in the Tree of Life, it is a suitable candidate marker to use in phylogenetic studies, likely beyond its application in bifidobacteria. In addition to being conserved genes that capture genetic diversity, glycolysis genes are consistently amongst the most highly expressed in not only *Bifidobacterium* (Turroni et al., 2015), but other organisms as well (Barrangou et al., 2006). This reflects both the importance of these sequences genetically (as illustrated by GC content drift), and functionally (as illustrated by their propensity for high levels of constitutive transcription). Because of this, it may be possible to correlate transcriptional data to phylogenetic studies on a broader scale. From here, it could be feasible to assign species and map data to known references using transcriptomic, genomic, or meta-data. Indeed, as the democratization of metagenomic technologies continues, and the need to assign phylogenetic information to partial genomic information increases, we propose that this method be used to provide insights into the phylogeny of un-assigned contigs. Overall, this approach allows for accurate phylogenetic mapping, congruent with a core-genome and more robust than the 16S rRNA phylogenetic approach, as well as inference on genomic adaptation, using either genomic, transcriptomic, or meta-data in a timely fashion and with minimal computation.

## Author Contributions

KB and RB designed and carried out experiments, interpreted results, and wrote the manuscript.

## Conflict of Interest Statement

The authors declare that the research was conducted in the absence of any commercial or financial relationships that could be construed as a potential conflict of interest.
