# Supplementary material for: Phylogenetic Analysis of the Bifidobacterium Genus Using Glycolysis Enzyme Sequences
Source: Front Microbiol. 2016 May 9;7:657. doi: 10.3389/fmicb.2016.00657 (PMC4860490; doi:10.3389/fmicb.2016.00657)
Supplement: Supplementary file 1 [file Image_1.PDF]

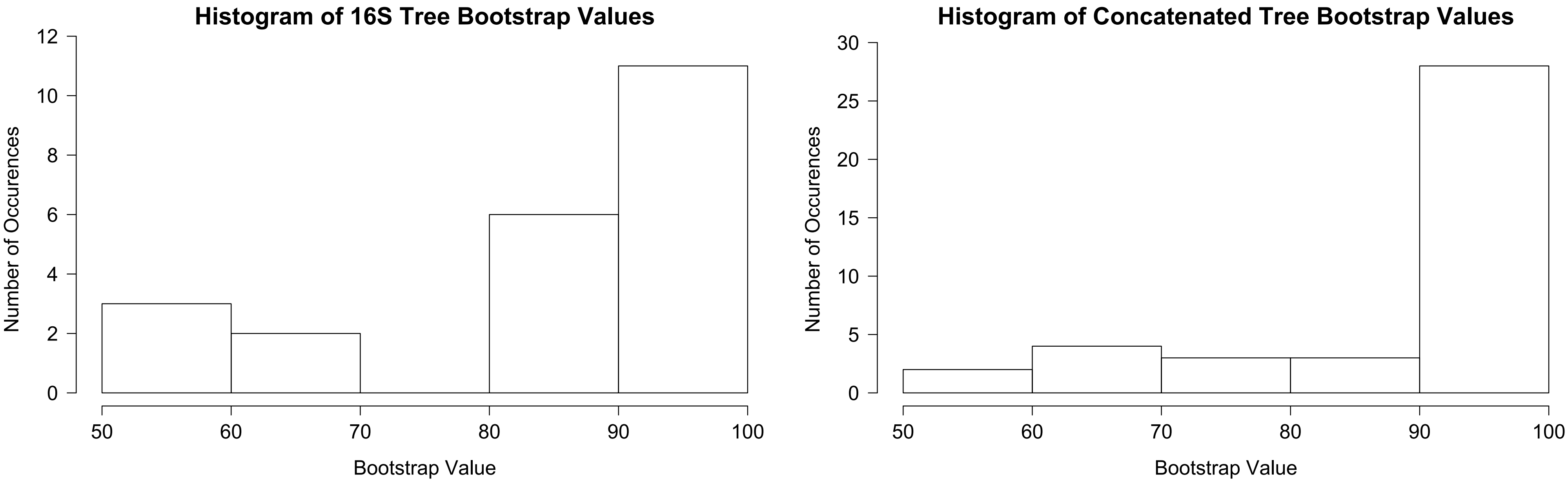

Supplemental Figure 1. Histogram of Bootstrap Values. Histograms of bootstrap values from the consensus trees of the 16S phylogenetic tree (right) and the glycolytic proteins concatenated tree (left).
