# Supplementary material for: Phylogenetic Analysis of the Bifidobacterium Genus Using Glycolysis Enzyme Sequences
Source: Front Microbiol. 2016 May 9;7:657. doi: 10.3389/fmicb.2016.00657 (PMC4860490; doi:10.3389/fmicb.2016.00657)
Supplement: Supplementary file 6 [file Image_6.PDF]

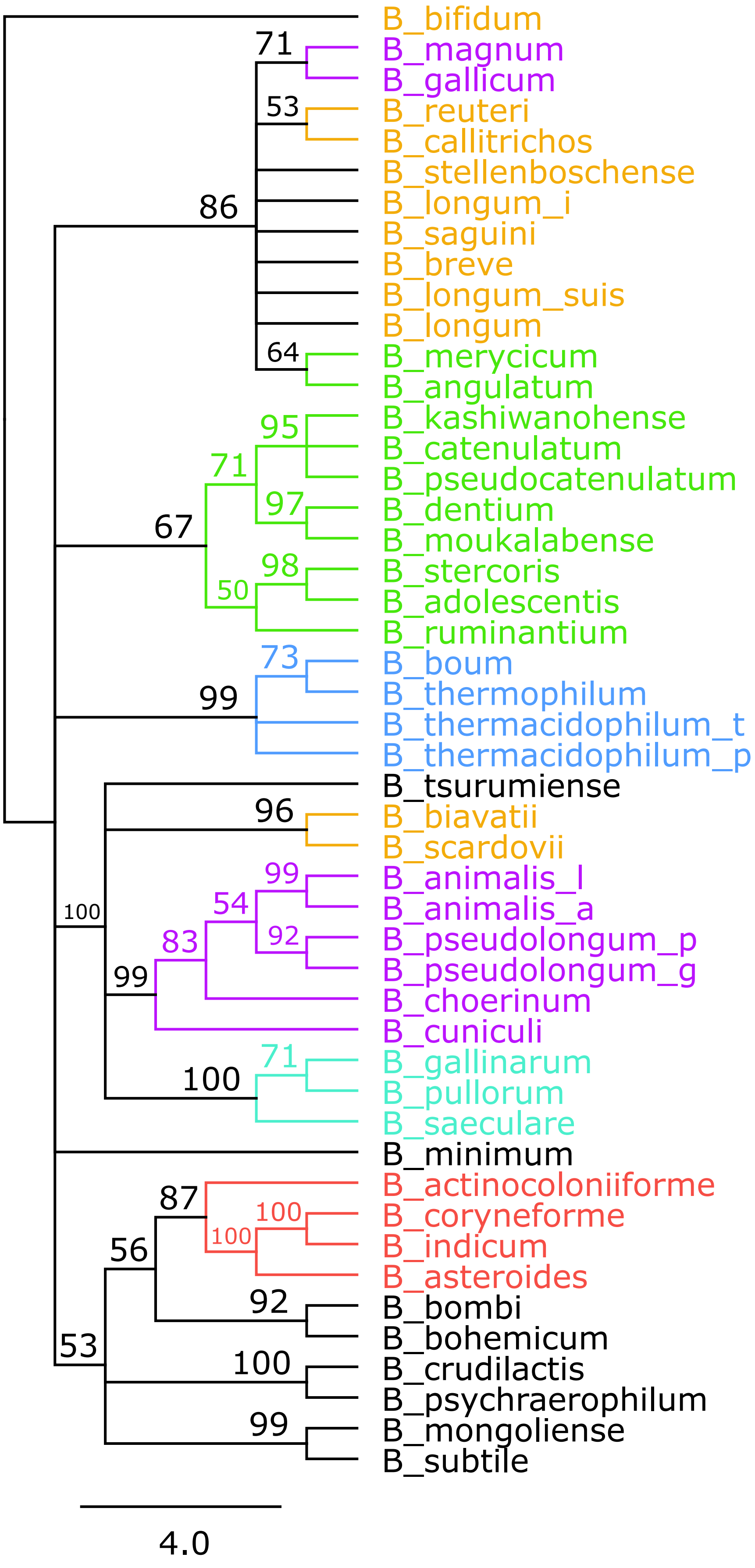

Supplemental Figure 6. Gap Tree. Consensus tree based on alignment of the amino acid sequences of Gap. Trees were made using RaxML. Bootstrap values are found on each node. Phylogenetic groups are colored as follows: *Bifidobacterium longum* is orange, *Bifidobacterium adolescentis* is green, *Bifidobacterium psdeudolongum* is purple, *Bifidobacterium pullorum* is blue-green, *Bifidobacterium boum* is blue, and *Bifidobacterium asteroides* red. Species names following the naming convention from Table 1.
